# Associations of the serum kynurenine pathway metabolites with P50 auditory gating in non-smoking patients with first-episode schizophrenia

**DOI:** 10.3389/fpsyt.2022.1036421

**Published:** 2022-10-20

**Authors:** Qingyan Yang, Yong Zhang, Kebing Yang, Yajuan Niu, Fengmei Fan, Song Chen, Xingguang Luo, Shuping Tan, Zhiren Wang, Jinghui Tong, Fude Yang, Chiang-Shan R. Li, Yunlong Tan

**Affiliations:** ^1^Peking University Huilongguan Clinical Medical School, Beijing Huilongguan Hospital, Beijing, China; ^2^Department of Psychiatry, Yale University School of Medicine, New Haven, CT, United States; ^3^Department of Neuroscience, Yale University School of Medicine, New Haven, CT, United States

**Keywords:** first-episode schizophrenia, kynurenine pathway, kynurenine, kynurenine acid, sensory gating, P50

## Abstract

**Objective:**

Our study aimed to investigate the associations between the serum level of kynurenine pathway (KP) metabolites and P50 auditory gating in non-smoking patients with first-episode schizophrenia (FES).

**Materials and methods:**

In this study, 82 non-smoking patients with FES and 73 healthy controls (HC). P50 auditory gating was measured using a fully functional digital 64-channel EEG system, and the components included S1 amplitude, S2 amplitude, gating ratio (S2/S1), and amplitude difference (S1–S2). Serum levels of kynurenine and kynurenine acid were assessed using a combination of liquid chromatography with tandem mass spectrometry. Psychopathology was assessed by the Positive and Negative Syndrome Scale (PANSS).

**Results:**

The serum kynurenine (251.46 ± 65.93 ng/ml vs. 320.65 ± 65.89 ng/ml, *t* = –6.38, *p* < 0.001), and kynurenine acid levels (5.19 ± 2.22 ng/ml vs. 13.26 ± 4.23 ng/ml, *t* = –14.73, *p* < 0.001), S1 amplitude [2.88 (1.79, 3.78) μV vs. 3.08 (2.46, 4.56) μV, *Z* = –2.17, *p* = 0.030] and S1–S2 [1.60 (0.63, 2.49) μV vs. 1.92 (1.12, 2.93) μV, *Z* = –2.23, *p* = 0.026] in patients with FES were significantly lower than those in HC. The serum kynurenine and kynurenine acid levels were negatively associated with S1–S2 (*r* = –0.32, *p* = 0.004 and *r* = –0.42, *p* < 0.001; respectively) and positively correlated with S2/S1 ratio (*r* = 0.34, *p* = 0.002 and *r* = 0.35, *p* = 0.002; respectively) in patients.

**Conclusion:**

Our findings suggested that neuroactive metabolites of the KP might play an important role in sensory gating deficit in first episode patients with schizophrenia. Furthermore, metabolites of the KP may be a new target for the treatment of cognitive impairments in schizophrenia.

## Introduction

Cognitive impairment is one of the core symptoms of schizophrenia that mostly leads to social disabilities (1, 2) and determines the prognosis of the disease (3). As an essential component of human cognitive function, sensory gating refers to the neurological process of filtering out redundant or unnecessary sensory input during information processing (4), its dysfunction has been frequently observed in patients with schizophrenia (5). Individuals with schizophrenia show sensory gating deficits, which may be associated with impaired sustained attention (6), slowed processing speed (7), and poor working memory performance (7, 8).

Some studies suggested that sensory gating deficits might be associated with schizophrenia symptom severity (5). However, a systematic review reported that cognitive impairment was presented before the onset of first-episode schizophrenia (FES) and persisted even if psychotic symptoms have been normalized (9). Furthermore, previous studies have shown that sensory gating deficit exacerbated schizophrenia symptoms (10, 11). Sensory gating deficit, usually reflected by P50 suppression, is a sensitive biological marker for identifying schizophrenia (12, 13). However, these arguments have mainly been based on studies conducted in antipsychotic medication patients. Although there is some evidence supporting sensory gating deficits in individuals with schizophrenia, further studies are needed to assess the deficits according to different stages of the disease, symptom severity, treatment, and other possible confounders, such as smoking.

Growing evidence indicated that dysregulation of kynurenine pathway (KP) metabolites might play an important role in the pathophysiology of schizophrenia (14). Kynurenine (KYN) is a tryptophan metabolite that can cross the blood-brain barrier (BBB). Kynurenic acid (KYNA), a product of the KYN pathway of tryptophan metabolism, may cause glutamate hypofunction through sustained N-methyl D-aspartate (NMDA) receptor antagonism, which may cause psychotic symptoms and cognitive deficits in people with schizophrenia (15, 16). Interestingly, due to its antagonistic action on NMDA receptors, effective antioxidant properties and hydroxyl radical scavenging capacity, a highly elevated level of KYNA has also been found to have neuroprotection potential in experimental animals (17, 18). In contrast to the replicable findings that cerebrospinal fluid (CSF) levels of KYN and KYNA were significantly higher in patients with schizophrenia than in healthy controls (HC) (19), either increase (20) or decrease (21) in their serum and plasma levels has been found in patients with schizophrenia compared with that in controls. In addition, Shovestul et al. (22) found no differences in serum KYN and KYNA levels between patients with schizophrenia and controls.

As a glutamate receptor antagonist, KYNA is reported to inhibit α7-nicotinic acetylcholine receptors (α7nAChRs) and may also act on targets downstream of α7nAChRs function (23). Recent studies have suggested that deficits in auditory gating are associated with α7nAChRs dysfunction (24). Preclinical research has revealed a link between elevated brain KYNA levels and disruption of auditory gating in adult rodents (25, 26). However, correlations between auditory gating deficits and serum KYN and KYNA levels in people with schizophrenia remain unclear.

The aims of this study included, (1) to compare P50 auditory gating and serum KYN and KYNA levels between non-smoking patients with FES and HC; (2) to explore the association between P50 auditory gating and serum KYN and KYNA levels in patients with FES and HC, respectively. Our findings might provide evidence for understanding of neurobiological mechanisms and the treatment of schizophrenia.

## Materials and methods

### Participants

Eighty-two non-smoking patients with FES were enrolled from Beijing Huilongguan Hospital. All patients met the following inclusion criteria: Diagnostic and Statistical Manual of Mental Disorders fourth edition (DSM-IV), criteria for schizophrenia (< 3 years of illness duration); between 18 and 45 years; treated with antipsychotic medication for less than 2 weeks; and no major physical illness. Seventy-three HC were recruited through advertising from the local community and screened by a trained psychiatrist, excluding those with major psychiatric disorders, personality disorders, substance abuse, major medical conditions, and a history of traumatic brain injury. All participants had normal hearing acuity (subjective hearing threshold of 40 dB) and were non-smokers, given that hearing acuity and smoking may affect auditory sensory gating and the kynurenine pathway, respectively (27). All participants were Han Chinese. This study was approved by the Research and Ethics Committee of Beijing Huilongguan Hospital. All eligible participants signed consent forms and were informed of their right to withdraw consent at any time.

### Measures

#### P50 testing

The electroencephalography signals were recorded using a fully functional digital 64-channel electroencephalography system (Brain Products, Germany). We assessed sensory gating by recording P50 waves of auditory evoked responses in the conditioned reflex test paradigm. The test was conducted in a shielded sound-insulating room. The participants sat down and were asked to relax their entire body but remained awake and focused while awaiting stimulation.

Sensory gating is usually evaluated using neurophysiological approaches in the auditory double-click paradigm (28). The double-click paradigm involves two auditory stimuli, 500 ms apart, to measure the amplitude response. Data from the apex are reported because this is the best site to distinguish patients with schizophrenia from healthy participants when using this electrode array (29).

P50 inhibition was recorded in the conditional test mode. According to the international electroencephalography 10/20 system, the recording electrode is placed in the central midline, the reference electrode is placed in the right ear, the forehead is grounded, and the impedance between the electrodes is set at < 5 K. The analysis time was 600 ms, non-target stimulation frequency was 1,000 Hz, and rule intensity was 80 dB. The target stimulation frequency was 4,000 Hz, probability was 20%, and intensity was 90 dB. It appears randomly and is mixed with non-target stimulation.

The regulatory P50 wave (S1) was the most positive peak between 40 and 90 ms after regulatory stimulation. The test P50 wave (S2) was determined as the maximum peak after test stimulation, which was closest to the regulated P50 wave during the incubation period. In the present study, P50 auditory gating components included the S1 amplitude, S2 amplitude, amplitude difference (S1–S2), and gating ratio (S2/S1) (5). S1 is an individual’s response to normal auditory stimuli, whereas S2 refers to the ability to suppress non-target stimuli in the presence of S1. The relative decrease in the P50 waveform from the first click (S1) to the second (S2) is typically used as an indicator of P50 auditory gating and quantified as the S1–S2 amplitude difference or the S2/S1 ratio. A lower ratio or higher P50 difference indicated stronger sensory gating (30).

#### Serum kynurenine and kynurenic acid levels

Blood samples were collected between 7 a.m. and 8 a.m. after overnight fasting, centrifuged at 3,000 rpm at 4°C for 10 min, and then the serum was separated and stored at –80°C. Serum KYN and KYNA levels were measured using high performance liquid chromatography and tandem mass spectrometry, and then quantified using standard protocols. Concentrations were calculated from the peak areas. The identity of the participants was coded and saved by the investigator until all biochemical analyses were completed.

#### Positive and Negative Syndrome Scale

Psychotic symptoms were assessed using the Positive and Negative Syndrome Scale (PANSS) (31). The PANSS is a 30-item rating assessment used to evaluate the severity of schizophrenia symptoms and contains three subscales: positive, negative, and general psychopathology. The raters were trained and showed good interrater reliability (intraclass correlation coefficient ≥ 0.75).

### Statistical analysis

Comparisons of serum levels of KYN and KYNA between patients with FES and HC were conducted using *t*-test and analysis of covariance (ANCOVA). Age and sex were controlled as possible confounding variables in the ANCOVA. Since the data of P50 parameters (S1 amplitude, S2 amplitude, S1–S2, and S2/S1 ratio) were highly skewed, the Mann–Whitney *U*-test was performed to compare differences between patients and HC. The χ^2^ test was performed to examine differences in categorical variables between the two groups.

Spearman’s correlations were used to test the associations between P50 parameters, serum KYN and KYNA levels, and the PANSS score in the patient group. We used spearman’s correlations to analyze the relationships between P50 parameters and serum KYN and KYNA levels in HC. Pearson’s correlations were used to analyze the associations between serum KYN and KYNA levels and PANSS scores. Two-tailed tests were performed for the analyses, with the significance level set at 0.05.

## Results

### Baseline clinical and demographic participant characteristics

Table 1 summarizes the demographic and clinical characteristics of all participants. There were no significant differences in age, sex, or educational level between the patient and control groups (*p* > 0.05). After inclusion in the study, 76 patients (92%) commenced treatment with antipsychotics (2 with first-generation and 74 with second-generation antipsychotics).

**TABLE 1:** Demographic and clinical characteristics of samples.

	Patient group (*n* = 82)	Control group (*n* = 73)	t/χ^2^	*p*
Male/Female	40/42	36/37	< 0.01	1.000
Age, years	27.95 ± 7.52	28.71 ± 6.07	–0.69	0.493
Years of education	13.66 ± 3.11	14.32 ± 2.08	–1.56	0.121
Age at onset, years	26.58 ± 7.56	NA	NA	NA
Duration of illness, months	6.36 ± 8.47	NA	NA	NA
PANSS total score	76.07 ± 11.93	NA	NA	NA
Positive symptoms	21.94 ± 5.04	NA	NA	NA
Negative symptoms	17.71 ± 5.54	NA	NA	NA
General psychopathology	36.42 ± 6.86	NA	NA	NA

NA, not applicable; PANSS, Positive and Negative Syndrome Scale.

### Comparisons of P50 parameters (S1, S2, S1–S2, and S2/S1) and serum kynurenine and kynurenic acid levels between patient and control groups

The S1 amplitude and amplitude difference (S1–S2) were significantly lower in the patient group than in the control group (*Z* = –2.17, *p* = 0.030 and *Z* = –2.23, *p* = 0.026; respectively), while there was no difference in S2 amplitude and S2/S1 ratio between the two groups. Compared with the control group, patients had lower serum levels of KYN (*t* = –6.38, *p* < 0.001) and KYNA (*t* = –14.73, *p* < 0.001). These significant differences were still observed in ANCOVA analyses after controlling for age and sex (Table 2).

**TABLE 2:** Comparisons of P50 parameters and serum levels of KYN and KYNA between patients and healthy controls.

	Patient group (*n* = 82)	Control group (*n* = 73)	*t/Z*	*P-*value
Kynurenine (ng/ml)^a^	251.46 ± 65.93	320.65 ± 65.89	–6.38	**< 0.001**
Kynurenic acid (ng/ml)^a^	5.19 ± 2.22	13.26 ± 4.23	–14.73	**< 0.001**
**P50**				
S1 amplitude (μV)^b^	2.88 (1.79, 3.78)	3.08 (2.46, 4.56)	–2.17	**0.030**
S2 amplitude (μV)^b^	1.20 (0.63,1.97)	1.19 (0.81, 1.80)	–0.08	0.937
S1–S2 (μV)^b^	1.60 (0.63, 2.49)	1.92 (1.12, 2.93)	–2.23	**0.026**
S2/S1 ratio^b^	0.43 (0.24, 0.69)	0.41 (0.23, 0.52)	–1.14	0.178

^a^Values are presented as mean ± standard deviation.

^b^Values are presented as median (25th percentile, 75th percentile).

Bold values indicate a statistically significant difference with a *p*-value < 0.05.

### Correlations between the P50 parameters (S1, S2, S1–S2, and S2/S1), serum kynurenine and kynurenic acid levels, and psychotic symptoms

**FIGURE 1:**
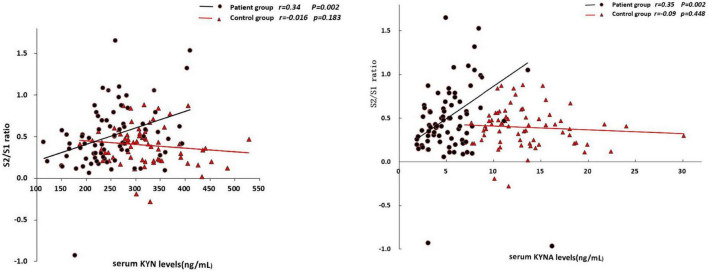
Correlations between S2/S1 ratio and the serum levels of KYN and KYNA, respectively.

**FIGURE 2:**
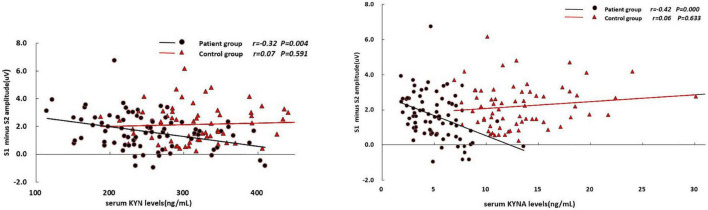
Correlations between S1–S2 amplitude difference and the serum levels of KYN and KYNA, respectively.

In the patient group, the serum KYN levels was positively associated with S2 amplitude (*r* = 0.24, *p* = 0.038; Table 3) and S2/S1 ratio (*r* = 0.34, *p* = 0.002; Figure 1), but negatively associated with amplitude difference (S1–S2) (*r* = –0.32, *p* = 0.004; Figure 2). There was no significant association between S1 amplitude and serum KYN levels (Table 3).

**TABLE 3:** Correlations between P50 parameters, the serum levels of KYN and KYNA as well as the severity of symptoms in the patient group.

	Kynurenine		Kynurenic acid		S1 amplitude		S2 amplitude		S1–S2		S2/S1	
	*r*	*P*	*r*	*p*	*r*	*p*	*r*	*p*	*r*	*p*	*r*	*p*
Kynurenine					–0.15	0.194	0.24	**0.038**	–0.32	**0.004**	0.34	**0.002**
Kynurenic acid					–0.31	**0.005**	0.12	0.303	–0.42	**< 0.001**	0.35	**0.002**
PANSS total score	–0.03	0.808	0.17	0.131	–0.10	0.391	–0.02	0.841	–0.09	0.418	0.06	0.607
Positive subscale	–0.04	0.711	0.11	0.348	–0.18	0.099	–0.03	0.784	–0.17	0.122	0.15	0.179
Negative subscale	–0.06	0.578	–0.10	0.374	0.21	0.062	0.04	0.708	0.11	0.339	–0.07	0.520
General psychopathology	0.03	0.773	0.30	**0.007**	–0.17	0.124	–0.07	0.519	–0.09	0.442	0.02	0.844

PANSS, Positive and Negative Syndrome Scale. Bold values indicate a statistically significant difference with a *p*-value < 0.05.

The serum KYNA level was positively associated with S2/S1 (*r* = 0.35, *p* = 0.002; Figure 1), but negatively associated with amplitude S1 (*r* = –0.31, *p* = 0.005; Table 3) and amplitude difference (S1–S2) (*r* = –0.42, *p* < 0.001; Figure 2). There was no significant correlation between serum KYNA level and amplitude S2 (Table 3).

Neither the P50 parameters nor serum KYN level was significantly associated with positive, negative, or general pathological symptoms. The serum KYNA level was significantly correlated with general pathological symptoms but not with positive or negative symptoms. P50 parameters and serum KYN and KYNA levels were not associated with antipsychotic status.

In the control group, none of the P50 parameters were significantly associated with serum levels of KYN or KYNA.

## Discussion

This study was designed to examine presence of the deficits in sensory gating and its association with kynurenine pathway metabolites among non-smoking patients with FES. Moreover, this study investigated the relationships between sensory gating deficits and serum KYN and KYNA. In the present study, people with FES showed lower levels of amplitude S1 and amplitude difference (S1–S2) than HC, whereas no differences were found in S2 or S2/S1 ratio between the two groups. Serum levels of KYN and KYNA were lower in the patient group than in the control group. Furthermore, the higher the serum levels of KYN and KYNA, the greater the impairment in sensory gating would be in patients with FES.

Our findings are consistent with those of previous studies on impaired P50 auditory gating in patients with FES (32). Specifically, we found that patients with FES had abnormal responses to the first stimuli and failure suppression of the S2 response (S1–S2), whereas no impairments were observed in the S2 and S2/S1 ratios. These findings are supported by previous research on sensory gating deficits in Chinese patients with FES (5). Compared with HC, patients showed lower S1 amplitude, which may be explained by less sensitivity to novel sound stimuli and, therefore, decreased reactivity. P50 sensory gating reflects a predominantly pre-attentional filtering mechanism that protects higher-order cognitive functions (33). Some researchers have proposed that the S1 amplitude largely determines the differences in sensory gating between patients and controls (34). A lower P50 amplitude difference (S1–S2) that has been suggested to be the most reliable index of sensory gating (35), represents a weaker suppression of an evoked response. The impaired mechanisms of sensory gating can result in overflow of irrelevant sensory input and may be associated with thought disorders and cognitive fragmentation (32). Our findings confirm that sensory gating is impaired in the early stages of schizophrenia (36). Previous studies have frequently reported an elevated P50 gating ratio (S2/S1) in patients with prodromal and FES (37). However, the reliability of the suppression ratio can be determined by the correlation between S1 and S2 (38). Although some studies (39) have demonstrated that more severe negative symptoms are related to more sensory gating deficits in schizophrenia, we failed to find any associations between P50 parameters and psychiatric symptoms.

Elevated levels of KYN and KYNA in the CSF and blood have often been reported in patients with schizophrenia, with or without antipsychotic treatment (20). Conversely, the present study found that the serum KYN and KYNA levels were significantly lower in patients than in HC. Our findings are in accordance with those of studies on individuals with first-episode and antipsychotic-naïve schizophrenia (40). Similarly, Szymona et al. (41) found reduced serum KYNA levels in patients with schizophrenia during acute relapse and after a 4-week treatment with neuroleptics. Antipsychotics have been found to possibly reduce peripheral KYN and KYNA in medication-naïve or medication-free patients with schizophrenia (42). In the present study, more than 90% of the patients were taking antipsychotic medication after they entered the study, which may have affected the results.

Previous studies (43) have found that inhibition of KYNA synthesis is associated with increased extracellular dopamine (DA) levels. The decreased KYNA level may lead to activation of α7nAChRs, which can trigger an increase in glutamate and DA release. On the other hand, elevated DA concentration may reduce KYNA production by interacting with astrocytic DA receptors (43, 44). Therefore, decreased levels of KYN and KYNA might represent a possible feedback mechanism following increased DA neurotransmission in schizophrenia (45). A meta-analysis (20) suggested elevated KYNA levels in patients with schizophrenia when measured in the central nervous system rather than in the periphery. Additionally, factors such as patients’ age, sex and antipsychotic treatment may affect the expression of KYN and KYNA in serum. In the present study, patients were in their late 20 s and had a mean illness duration of 6 months. The mean PANSS total score (76.07 ± 11.93) indicated that they were moderately to markedly ill (46). A recent meta-analysis (47) showed that the peripheral KYNA level was significantly decreased in patients with schizophrenia spectrum disorders, particularly those with acute and severe symptoms. Our results revealed that a higher serum level of KYNA was associated with more severe general psychopathology; however, no correlation was found between the serum levels of KYN and KYNA and age, sex, or other PANSS dimensions.

The associations between serum levels of KYN and KYNA and P50 parameters existed only in the patient group. In agreement with previous preclinical and clinical studies (48–50), our study showed that among non-smoking patients with FES, higher levels of KYN and KYNA were associated with more impairments in auditory gating, namely decreased amplitude differences and a higher P50 auditory gating ratio. KYNA is widely considered neuroprotective because of its capacity to block neuronal excitation (48); however, abnormally increased KYNA levels may play a role in impaired attention and vigilance among people with schizophrenia (49, 50). For example, a fourfold increase in brain KYNA levels was associated with reduced prepulse inhibition in rats, an effect mimicking clinical sensory gating impairment (26). Previous research has postulated that KYNA is a non-competitive antagonist of α7nAChRs (51). Furthermore, combined cholinergic treatment with galantamine, a positive allosteric modulator of α7nAChRs, increased and prolonged nicotinic receptor activity, alleviating P50 auditory gating impairments. These findings suggest that sensory gating deficits may be associated with α7nAChRs dysfunction (24). Hence, the association between serum levels of KYN and KYNA and P50 impairments might be mediated by α7nAChRs dysfunction. As a significant molecular and neuropharmacological target, α7nAChRs have been found to improve learning, memory, and attentional mechanisms. Moreover, they are essential modulators of sensory gating (52). Our findings suggest that treatment regulating the NMDAergic system and targeting α7nAChRs could potentially improve auditory sensory gating and cognitive function in people with schizophrenia (53).

This study design has several limitations. First, the cross-sectional design limited the inference of causality. Prospective and longitudinal studies should be conducted to clarify the causal relationship between kynurenine pathway metabolites and P50 sensory gating. Second, most patients began using antipsychotic treatment after entering this study, which may have affected kynurenine pathway metabolites and sensory gating function. However, antipsychotic status was not associated with KYN and KYNA levels or with P50 parameters in this study. Third, many unmeasured potential variables, such as distress intolerance (54) and childhood trauma (22), may influence serum levels of KYN and KYNA or sensory gating. Fourth, given our small sample size and non-normally distributed data, we were not able to conduct analyses of the functional relationship between sensory gating and serum levels of KYN and KYNA.

## Conclusion

To the best of our knowledge, the present study is the first to examine serum kynurenine metabolites and P50 sensory gating, as well as their association in non-smoking patients with FES. Compared to HC, patients with FES have sensory gating deficits showing lower S1 amplitudes, amplitude differences, and serum levels of KYN and KYNA. Higher serum KYN and KYNA levels were associated with more severe P50 gating deficits. Treatments targeting the kynurenine pathway, α7 nicotinic and NMDA receptors may improve the regulation of sensory gating performance in patients with FES.

## Data availability statement

The data that support the findings of this study are available at: https://www.jianguoyun.com/p/DV5YmLEQt66GCxi7xN8EIAA, further requests can be directed to yltan21@126.com.

## Ethics statement

The studies involving human participants were reviewed and approved by the Research and Ethics Committee of Beijing Huilongguan Hospital. The patients/participants provided their written informed consent to participate in this study.

## Author contributions

YT: concept and design, integrity of the data, accuracy of data analysis, full access to all data in the study, administrative, technical, and material support. QY and YZ: drafting of the manuscript. XL, ST, C-SL, and YT: critically revise manuscripts of important intellectual content. QY, YZ, FF, and YT: statistical analysis. YZ, SC, and YT: obtain the funding. All authors contributed to the acquisition, analysis, and interpretation of data.

## Abbreviations

KP: kynurenine pathway
KYN: kynurenine
KYNA: kynurenic acid
FES: first-episode schizophrenia
NMDA: N-methylD-aspartate
CSF: cerebrospinal fluid
α 7nAChRs: α 7-nicotinic acetylcholine receptors
PANSS: Positive and Negative Syndrome Scale
ANCOVA: analysis of covariance.
